# Bulked Segregant RNA-Seq Provides Distinctive Expression Profile Against Powdery Mildew in the Wheat Genotype YD588

**DOI:** 10.3389/fpls.2021.764978

**Published:** 2021-12-03

**Authors:** Pengtao Ma, Liru Wu, Yufei Xu, Hongxing Xu, Xu Zhang, Wenrui Wang, Cheng Liu, Bo Wang

**Affiliations:** ^1^School of Life Sciences, Yantai University, Yantai, China; ^2^School of Life Sciences, Henan University, Kaifeng, China; ^3^Crop Research Institute, Shandong Academy of Agricultural Sciences, Jinan, China

**Keywords:** wheat, powdery mildew, BSR-seq, expression profiling, DEG

## Abstract

Wheat powdery mildew, caused by the fungal pathogen *Blumeria graminis* f. sp. *tritici* (*Bgt*), is a destructive disease leading to huge yield losses in production. Host resistance can greatly contribute to the control of the disease. To explore potential genes related to the powdery mildew (*Pm*) resistance, in this study, we used a resistant genotype YD588 to investigate the potential resistance components and profiled its expression in response to powdery mildew infection. Genetic analysis showed that a single dominant gene, tentatively designated *PmYD588*, conferred resistance to powdery mildew in YD588. Using bulked segregant RNA-Seq (BSR-Seq) and single nucleotide polymorphism (SNP) association analysis, two high-confidence candidate regions were detected in the chromosome arm 2B, spanning 453,752,054-506,356,791 and 584,117,809-664,221,850 bp, respectively. To confirm the candidate region, molecular markers were developed using the BSR-Seq data and mapped *PmYD588* to an interval of 4.2 cM by using the markers *YTU588-004* and *YTU588-008*. The physical position was subsequently locked into the interval of 647.1–656.0 Mb, which was different from those of *Pm6*, *Pm33*, *Pm51*, *Pm52*, *Pm63*, *Pm64, PmQ*, *PmKN0816*, *MlZec1*, and *MlAB10* on the same chromosome arm in its position, suggesting that it is most likely a new *Pm* gene. To explore the potential regulatory genes of the *R* gene, 2,973 differentially expressed genes (DEGs) between the parents and bulks were analyzed using gene ontology (GO), clusters of orthologous group (COG), and Kyoto Encyclopedia of Genes and Genomes (KEGG) pathway enrichment analysis. Based on the data, we selected 23 potential regulated genes in the enriched pathway of plant-pathogen interaction and detected their temporal expression patterns using an additional set of wheat samples and time-course analysis postinoculation with *Bgt*. As a result, six disease-related genes showed distinctive expression profiles after *Bgt* invasion and can serve as key candidates for the dissection of resistance mechanisms and improvement of durable resistance to wheat powdery mildew.

## Introduction

Common wheat (*Triticum aestivum* L.) is one of the most important cereal crops worldwide (Hoisington et al., 1999). However, powdery mildew, caused by *Blumeria graminis* f. sp. *tritici*, (*Bgt*), can lead to huge yield losses (Everts et al., 2001; Singh et al., 2016; Tsai et al., 2020). To control this disease, farmers traditionally relied on fungicide. But the pathogenic bacteria were easily evolved variation and produced drug resistance due to the overuse of pesticides (Twamley et al., 2019). In addition, environmental pollution along with the use of pesticides become increasingly prominent (Felsenstein et al., 2010). Comparatively speaking, host resistance is generally considered to be the most economical, sustainable, and environmentally friendly approach to control wheat powdery mildew (Johansson et al., 2020; Li Y. et al., 2021).

To improve host resistance, the *R* genes were commonly identified and used in breeding. To date, more than 100 formally designated powdery mildew resistance (*Pm*) genes at 63 loci are reported in common wheat and its relatives (Li G. et al., 2019; Maccaferri et al., 2019; Tan et al., 2019; Li Y. et al., 2020; Zhu et al., 2020; Wu et al., 2021). These resistance genes could be classified into two categories: qualitative resistance and quantitative resistance. Most reported *Pm* genes belonged to qualitative resistance and follow Mendel’s law of segregation (Ma et al., 2014; Zhu et al., 2020). Owing to their high resistance to different virulent isolates, qualitative resistance genes have been widely utilized in wheat cultivars, such as *Pm8* and *Pm21* (Hurni et al., 2014; Xing et al., 2018; Li H. et al., 2020; Wu et al., 2021). Compared with quantitative resistance, qualitative resistance is often defeated due to the shifts of *Bgt* virulence in wheat production (Xiao et al., 2013). Thus, it is urgent to discover new *Pm* genes/alleles and dissect the molecular mechanism of resistance, which is valuable for durable resistance in production.

Due to the large genome (exceed 17 Gb) and allohexaploid property, exploring and dissecting genetic traits of the allohexaploid wheat is difficult (Gupta et al., 2008; Wu et al., 2018). Efficient methods are needed to detect single nucleotide polymorphism (SNP), map the *R* gene, and assess the *Pm* gene expression profiles and describe the differential gene expression profiling. In recent years, whole-genome sequencing of common wheat and its relatives has made great progress, and corresponding high-throughput sequencing techniques have been greatly improved and used to construct linkage maps and dissect the molecular expression pattern of functional genes. Especially, bulked segregant analysis-RNA-Seq (BSR-Seq), combined RNA-Seq, and bulked segregant analysis (BSA) are advanced strategies for both holistic studies of the expression profile of complex genome and gene/quantitative trait locus (QTL) mapping (Michelmore et al., 1991; Pearce et al., 2015; Pankievicz et al., 2016). According to this method, mutant candidate genes and a large amount of SNP markers can be identified, and subsequently, comparative genomics analyses and high-density genetic linkage maps can also be carried out (Liu S. et al., 2012; Trick et al., 2012; Li L. et al., 2014; Ramirez-Gonzalez et al., 2014; Wang et al., 2017; Wu et al., 2018; Zhu et al., 2020).

Meanwhile, plants’ resistance to biotic stresses is an extraordinarily complex process. In the long-term interactions between plant and pathogen, plants evolved two layers of innate immunity: the PAMP-triggered immunity (PTI) pathway and the effector-triggered immunity (ETI) pathway (Jones and Dangl, 2006). The PTI is the first layer of the plant immunity system, where cells receive and transduce extracellular stress signals into the intracellular environment. Many receptor-like protein kinases (RLKs) especially leucine-rich repeat (LRR)-RLKs act as sensors and receptors in PTI (Chinchilla et al., 2007; Miya et al., 2007; Postel et al., 2010; Liu T. et al., 2012; Schwessinger and Ronald, 2012). The ETI is triggered by effectors injected into plant cells from pathogens and is often associated with an hypersensitive response (HR) (Jones and Dangl, 2006; Liu C. et al., 2019). In the whole process of plant resistance to pathogenic microorganisms, many factors play direct or indirect roles, including mitogen-activated protein (MAP) kinase signaling cascade system (Asai et al., 2002; He et al., 2006; Gao et al., 2008; Galletti et al., 2011), plant hormone signaling associated proteins (Navarro et al., 2008; Millet et al., 2010; Kim et al., 2014; Naseem et al., 2015), calcium influx (Kwaaitaal et al., 2011), nucleotide-binding LRR (NB-LRR) protein (Zhang et al., 2012), other kinases (Yamada et al., 2016), and transcription factors (Sarris et al., 2015) and so on. However, the immune mechanism in host resistance mainly focuses on the model plant *Arabidopsis.* Few studies reported the pathogen resistance mechanism in common wheat with a complex genome.

YD588, a Chinese wheat breeding line, exhibits high-level resistance to powdery mildew at both seedling and adult stages. In order to rapidly confirm the genetic model, candidate interval of the *R* gene(s), and profile the expression of resistance-related regulatory genes, using BSR-Seq, we (1) conformed the candidate interval of the *R* gene by analyzing the distribution of SNPs and molecular mapping and (2) profiled the key regulatory genes that may mediate powdery mildew resistance following *Bgt* inoculation.

## Materials and Methods

### Plant Materials

The wheat breeding line YD588 was used as the resistant parent against powdery mildew and the susceptible cultivar Yannong 21 (YN21) was used as susceptible to be crossed with YD588 in order to produce F_1_. For the F_2_ and F_2__:__3_ families, 210 F_2_ plants and 205 generated F_2__:__3_ families were harvested for analyzing the inheritance of the resistance gene and BSR-Seq. Wheat cultivar Huixianhong was used as the susceptible control in evaluating the powdery mildew resistance. Twenty-six wheat genotypes with documented *Pm* genes were used to evaluate the powdery mildew resistance in YD588.

### Evaluation of Powdery Mildew Response to *Bgt* Isolates

To evaluate the host resistance, YD588 was tested against 16 *Bgt* isolates with different virulence using 26 wheat genotypes with documented *Pm* genes as controls. To confirm the inheritance of the host resistance in YD588, YD588, YN21, and their derived F_1_, F_2_, and F_2__:__3_ progenies were inoculated with the *Bgt* isolate Y03 for phenotypic assessment at the one-leaf stage. The test seedlings were infected using the dusting method based on the previous studies (Ma et al., 2014). Seedlings to be evaluated were grown in a high humidity environment at 18°C/12°C (day/night) with a 12/14 h photoperiod. The test seedlings were inoculated with fresh conidiospores previously amplified on Huixianhong seedlings, and immediately incubated in a dark and 100% humidity space at 18^*o*^C for 24 h, and then the growth chamber was set to 20^*o*^C with a daily photoperiod of 14 h. For the next two days, the above inoculation process was repeated two times in the dark. When the spores were fully developed on the first leaf of the susceptible control Huixianhong (about 10–14 days after inoculation), infection types (ITs) were scored according to a 0–4 scale based on the previous studies (Si et al., 1992; An et al., 2013). The IT scores of 0–2 were considered as resistant type, while the scores 3 and 4 were considered as susceptible type. Under the same procedure, three parallel experiments were conducted to confirm the phenotype.

### Samples Preparation for Bulked Segregant RNA-Seq

For YD588, YN21, and their derived F_1_ hybrids, 10 seeds of each genotype were sown. For the F_2_ population and F_2__:__3_ families, 210 F_2_ plants and 205 F_2__:__3_ families (20 seeds for each family) were sown for genetic analysis and preparation of the samples for BSR-Seq. As susceptible control, Huixianhong was planted randomly in each tray. When the spores were fully developed on the first leaf of Huixianhong, the first leaves of two patents YD588 and YN21, 40 resistant plants from 40 homozygous-resistant F_2__:__3_ families, and 40 susceptible plants from 40 homozygous-susceptible F_2__:__3_ families were sampled. Using the Spectrum Plant Total RNA kit (Sigma-Aldrich, Shanghai), RNA from the above materials was extracted following the protocol of the manufacturer. Resistant and susceptible RNA bulks were pooled by separately mixing equal amounts of mRNA from the 40 homozygous resistant and susceptible F_2__;__3_ plants, respectively. The mRNA of YD588, YN21, resistant bulks, and susceptible bulks were used for subsequent RNA-Seq.

### Library Construction and RNA Sequencing

Using RNA samples with Integrity Number (RIN) ≥ 7, cDNA libraries of YD588, YN21, and the resistant and susceptible bulks were constructed. RNA integrity assessment and cDNA libraries construction were performed based on the previous studies (Zhu et al., 2020). Then, using the Agilent 2100 Bioanalyzer (Agilent Technologies, Santa Clara, CA, United States), the quality of the cDNA libraries was assessed. The cDNA libraries sequencing was carried out on the Illumina HiSeq sequencing platform (Illumina HiSeq4000) by Beijing Biomics Technology Company Limited (Beijing, China). High-quality clean data were obtained after raw data filtering, sequences joint, and poor-quality reads elimination. The 10 and 20 Gb clean data were set for parents and bulks, respectively, in the sequencing indicator. Following mapping, the clean data to the reference genome of Chinese Spring (v1.1),^1^ SNP calling, DEG analysis, and gene ontology (GO) and Kyoto Encyclopedia of Genes and Genomes (KEGG) pathway analyses were performed in Cloud Platform developed by Beijing Biomics Technology Company Limited.

### Single Nucleotide Polymorphism Calling and Bulked Segregant RNA Association Mapping

After aligning the clean reads of YD588, YN21, and resistance and susceptible bulks with the Chinese Spring reference genome (IWGSC RefSeq, version 1.1; The International Wheat Genome Sequencing Consortium [IWGSC], 2018) using the STAR (version 2.3.0e; International Rice Genome Sequencing Project, and Sasaki, 2005), SNP calling was carried out by using the GATK software (version 3.1-1; McKenna et al., 2010) following the reference flowchart aimed at RNA-Seq. SNP index values were calculated using the SNPs in YN21 as a reference using the MutMap method (Abe et al., 2012). Subsequently, the ΔSNP index between resistant and susceptible parents and bulks for each SNP was calculated (Takagi et al., 2013) using the following formula:

ΔSNP_index = (SNP_index of resistance parent/bulk) - (SNP_index of susceptible parent/bulk).

Using a 5-Mb size as a step, the average value of ΔSNP index in each window was calculated by sliding the window. The threshold for SNP screening was set as a test of 100,000 permutations coupling with 99.0% confidence (Takagi et al., 2013). Candidate regions with higher confidence (exceeding 99.0%) and SNPs with larger than threshold ΔSNP index value (set as 0.75) in candidate regions were considered to be candidate loci related to powdery mildew resistance in YD588.

To further verify the result of the ΔSNP index, the Euclidean distance (ED) algorithm was also used to calculate the candidate region based on the method of Trapnell et al. (2010).

### Development of Genetic Markers and Molecular Mapping

To further locate and validate the interval of the *Pm* gene(s) in YD588, simple sequence repeat (SSR) in the candidate intervals obtained from the BSR-Seq were used to develop molecular markers, which were then tested for polymorphisms between YD588, YN21, and their derived resistant and susceptible bulks. The resulting markers were then genotyped on the segregation population of YD588 × YN21. PCR amplification, separation, and visualization of the PCR products were done using the method by Ma et al. (2014). After genotyping, the χ^2^ test was carried out to evaluate the deviation of the observed phenotypic data from theoretically expected segregation ratios for the goodness-of-fit analysis. The linkage map of the *Pm* gene(s) in YD588 was finally constructed using the MAPMAKER 3.0 and the Kosambi function based on the studies by Lincoln et al. (1993) and Kosambi (1943).

### Differentially Expressed Genes Analysis

After SNP calling, fragments per kilo bases of exon per million fragments mapped (FPKM) was used to calculate the expression level of functional genes mapped to the reference genome of Chinese Spring (version 1.1) (Garber et al., 2011). DEGs were defined as fold change ≥ 2 and false discovery rate (FDR) < 0.01 using the EBSeq software (Leng et al., 2013). Statistical significance of DEGs was determined using a combination of multiple tests and FDR (Reiner et al., 2003). The statistic and cluster analysis of DEGs between resistance and susceptible parents and bulks were performed, and then differential expression patterns in the whole genome including the candidate interval was presented.

### Functional Annotation and Enrichment Analysis of the Differentially Expressed Genes

Functional annotation of DEGs was performed based on the information from IWGSC RefSeq (version 1.1; The International Wheat Genome Sequencing Consortium [IWGSC], 2018). Then, GO, clusters of orthologous group (COG), and KEGG pathway enrichment analysis of the DEGs were implemented using the R package based on the previous studies (Yu et al., 2012). Tools and databases used in the GO, COG, and KEGG pathway analysis, respectively, were as follows: GO Term Finder (Boyle et al., 2004), UniGene sequences,^2^ and KEGG database.^3^

### Quantitative Reverse Transcription PCR

Differentially expressed genes used in the key signal pathway about disease resistance or stress tolerance were selected to profile their expression patterns *via* quantitative reverse transcription (qRT)-PCR, using an additional set of wheat samples and time-course analysis postinoculation with *Bgt*. The first leaves of YD588 and YN21 seedlings were sampled at 3, 6, 12, 24, 36, 48, and 72 h after inoculation with the *Bgt* isolate Y03. The collected leaves were immediately frozen in liquid nitrogen and ground to a fine powder in a pestle and mortar. Total RNA was extracted using RNAiso Plus, Beijing (TaKaRa Cat#9109) following the instruction manual of the manufacturer and quantified by measuring absorbance using a NanoDrop 1000 spectrophotometer (Thermo Scientific, Beijing). cDNA was synthesized from total RNA using the Fastking kit (Tiangen, KR118-02, Beijing).

The qRT-PCR analysis was performed using SYBR Green Master Mix (Tiangen) based on the previous studies (He et al., 2016, 2018). Amplification was followed by melt curve analysis. The 2^–ΔΔ*Ct*^ method was used for relative quantification (He et al., 2018). Primers for specific genes were designed based on the coding sequences of the selected genes. The housekeeping gene *Tubulin* was used for normalization. Three parallel experiments were set up.

## Results

### Resistance Evaluation and Genetic Analysis for the Powdery Mildew Resistance

**FIGURE 1:**
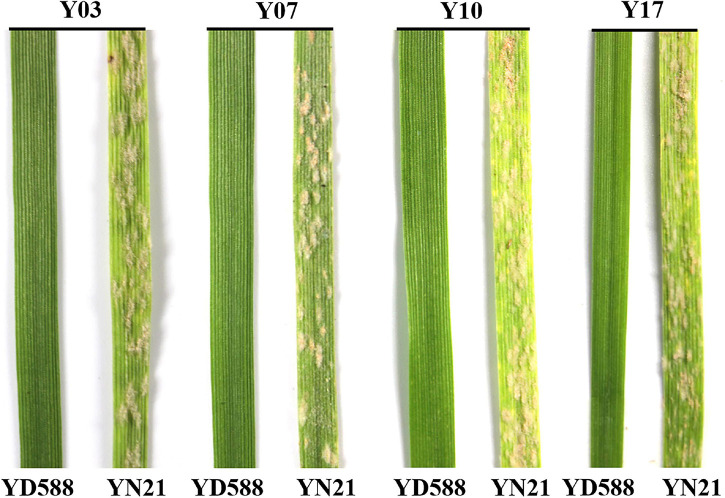
Seedling reaction patterns of YD588 and Yannong 21 (YN21) to the selected four *Blumeria graminis* f. sp. *tritici* (*Bgt*) isolates collected from different regions of China.

YD588 was resistant to 14 of the 16 *Bgt* isolates, accounting for a proportion of 87.5% (Figure 1 and Supplementary Table 1). Compared with the documented *Pm* genes, especially the *Pm2* and *Pm4* that were mainly used in the production in China, YD588 showed a higher potential in resistance breeding. Then, the *Bgt* isolate Y03 was selected to analyze the inheritance of the powdery mildew resistance in YD588. When tested against Y03, YD588 had no visible symptoms and displayed high resistance to *Bgt* when the IT score is 0. In contrast, over 80% of the leaf area was covered with aerial hyphae in YN21 and was considered to be highly susceptible when the IT score is 4. Subsequently, F_1_, F_2_, and F_2__:__3_ seedlings crossed by YD588 and YN21 were also inoculated with the same isolate. All the 10 tested F_1_ plants showed the same resistance pattern as YD588, suggesting a dominant inheritance pattern. The 210 tested F_2_ plants segregated into 155 resistant and 55 susceptible ones, following the theoretical ratio for monogenic segregation (χ^2^ = 0.16; *P* = 0.69). Subsequently, 205 generated F_2__:__3_ families harvested from the 210 tested F_2_ plants (five F_2_ plants died and cannot harvest F_3_ seeds) further confirmed the ratio for monogenic segregation, with a segregation ratio of 53 homozygous resistant (RR): 98 segregating (Rr): 54 homozygous susceptible (rr) families (χ^2^ = 0.40; *P* = 0.82). In conclusion, a single dominant gene, tentatively designated *PmYD588*, controls the powdery mildew resistance in YD588.

### Clean Data, Quality Control, and Sequence Alignments

After filtering low-quality data, adapter reads, and rRNA, a total of 13.35, 10.43, 28.59, and 24.87 Gb clean data for YD588, YN21, resistant, and susceptible bulks, respectively, were obtained, containing 44,657,220, 34,873,857, 95,638,544, and 83,147,454 clean reads, respectively. All the clean reads from the four samples account for more than 98.99% of total raw data, with Q30 >94.84%, and the GC content ranges from 52.49 to 56.12%. Following alignment of the four sets of clean reads to IWGSC RefSeq (version 1.1; The International Wheat Genome Sequencing Consortium [IWGSC], 2018) individually, 61.88–86.43% reads were mapped on the reference sequence (Supplementary Table 2). In summary, we obtained high-quality sequence data suitable for the subsequent analysis.

### Single Nucleotide Polymorphism Calling and Analysis of the Candidate Interval for Powdery Mildew Resistance

**FIGURE 2:**
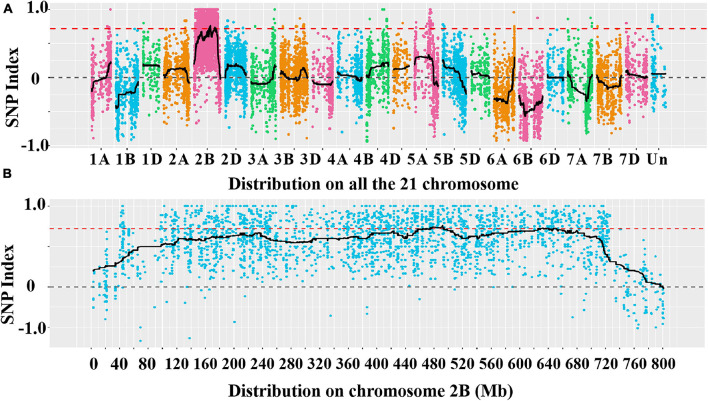
Distribution of the single nucleotide polymorphisms (SNPs) with consistent differences between the resistant parent YD588 and susceptible parent Yannong 21 (YN21) and their derived bulked pools on 21 chromosomes **(A)** and on chromosome arm 2B **(B)** based on the ΔSNP index value.

**FIGURE 3:**
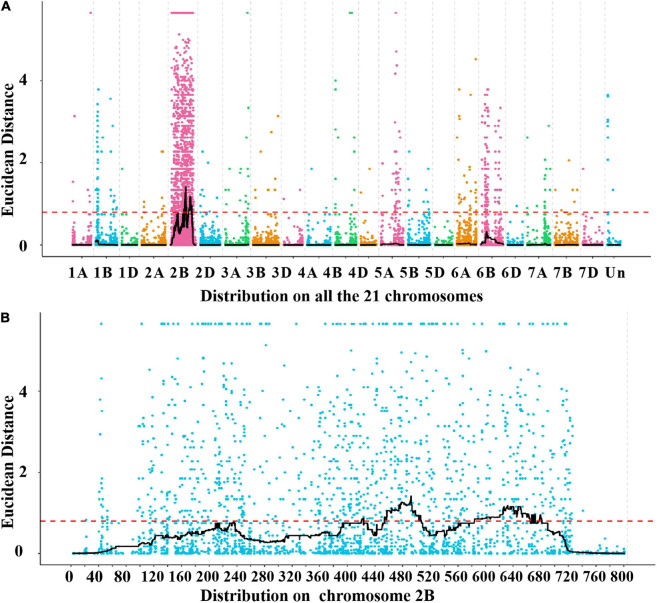
Distribution of the SNPs with consistent differences between the resistant parent YD588 and susceptible parent YN21 and their derived bulked pools on 21 chromosomes **(A)** and on the chromosome arm 2B **(B)** based on the Euclidean distance (ED) algorithm.

A total of 39,995 SNPs between parents and 50,644 SNPs between the bulks were detected, and 12,004 SNPs with consistent differences between resistant and susceptible parents and bulks were further obtained for the subsequent ΔSNP index analysis (Supplementary Table 3). Using the SNP index, six adjacent candidate regions on chromosome arm 2B were identified (Figure 2 and Table 1). To confirm the result, the ED algorithm was also performed, and similar results containing four adjacent candidate regions were obtained (Figure 3 and Table 2). Combining the two results, two high-confidence candidate regions were confirmed, spanning 453,752,054-506,356,791 and 584,117,809-664,221,850 bp, respectively.

**TABLE 1:** The candidate intervals of the *R* gene *PmYD588* and gene numbers in these intervals using SNP index.

**Chromosome_ID**	**Start (bp)**	**End (bp)**	**Size (Mb)**	**Gene_Number**
chr2B	234,639,633	236,047,412	1.41	7
chr2B	421,707,302	423,663,892	1.96	11
chr2B	453,752,054	506,356,791	52.60	478
chr2B	584,117,809	664,221,850	80.10	694
chr2B	667,264,262	669,442,164	2.18	21
chr2B	677,804,508	681,539,163	3.73	47

**TABLE 2:** The candidate intervals of the *R* gene *PmYD588* and gene numbers in these intervals using the Euclidean distance (ED) algorithm.

**Chromosome_ID**	**Start (bp)**	**End (bp)**	**Size (Mb)**	**Gene_Number**
chr2B	421,707,302	423,663,892	1.96	11
chr2B	453,752,054	506,356,791	52.60	478
chr2B	584,117,809	664,221,850	80.10	694
chr2B	667,264,262	669,442,164	2.18	21

### Molecular Mapping of *PmYD588*

**FIGURE 4:**
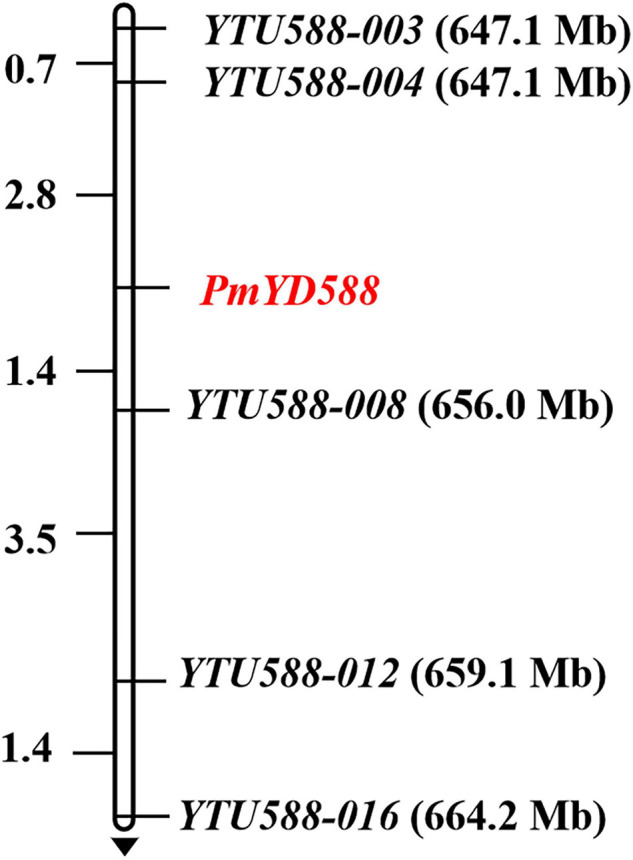
Linkage map of *PmYD588* using the F_2__:__3_ families of YD588 × YN21. Genetic distances in cM are showed to the left. The black arrows point to the centromere.

To map *PmYD588*, 18 SSR markers were developed using the BSR-Seq data. As a result, five markers (*YTU588-003*, *YTU588-004*, *YTU588-008*, *YTU588-012*, and *YTU588-016*) showed consistent polymorphism between the parents and bulks (Table 3). After genotyping the segregation population of YD588 and YN21, these markers were also linked with *PmYD588* (Figure 4). A linkage map was then constructed, and *PmYD588* was flanked by *YTU588-004* and *YTU588-008* with genetic distances of 2.8 and 1.4 cM, respectively. The corresponding physical interval was 647.7–656.0 Mb in the chromosome arm 2BL (Figure 4).

**TABLE 3:** Molecular markers that were newly developed using BSR-Seq for the powdery mildew resistance gene *PmYD588.*

**Marker**	**Location (bp)**	**Primer sequence**	**Marker type**
*YTU588-001-F*	chr2B_641097246	GAGTTCATCTGCCCGGACT	SSR
*YTU588-001-R*		TGACGAGAGGAGGTAATGCC	SSR
*YTU588-002-F*	chr2B_643299165	TATTTGCAGCGAGACAATGG	SSR
*YTU588-002-R*		GCTAGTGCTCCCAGGAACTG	SSR
*YTU588-003-F*	chr2B_647099942	GGCCAACGAACGAATGTAGT	SSR
*YTU588-003-R*		GTCAAACCAGGGGCTTCTCT	SSR
*YTU588-004-F*	chr2B_647107905	GGGTTGCTGCGTTTACTTGT	SSR
*YTU588-004-R*		TAACCAAATGTGTGGCAGGA	SSR
*YTU588-005-F*	chr2B_650284601	GGCAGCTAGATGCAATTAAGG	SSR
*YTU588-005-R*		GCCAATTACTCTCATGGGCA	SSR
*YTU588-006-F*	chr2B_650284610	AGATGCAATTAAGGTATGCCC	SSR
*YTU588-006-R*		TTTCCTTTTCCATGGCAATTA	SSR
*YTU588-007-F*	chr2B_651931942	CCAAGTCAGGCAGTAGCCTC	SSR
*YTU588-007-R*		CAGTCTGAAACCTTGTCGCA	SSR
*YTU588-008-F*	chr2B_655987001	GAGAACATCGCGAAAATGGT	SSR
*YTU588-008-R*		GCCCACGAATTTTAACTGGA	SSR
*YTU588-009-F*	chr2B_655987619	GAAAAGAGAGGCAGTTGCTGA	SSR
*YTU588-009-R*		GCCCACGAATTTTAACTGGA	SSR
*YTU588-010-F*	chr2B_657812694	AAGTTAATCCCACCAGCGTG	SSR
*YTU588-010-R*		GCTCGTCCTGACCGACTATC	SSR
*YTU588-011-F*	chr2B_658996691	ACGTGCACATGCTCTTCATC	SSR
*YTU588-011-R*		ATCAATAAAACCGTGGGTGC	SSR
*YTU588-012-F*	chr2B_659068425	TATTTGCGTGCATGGTTGAT	SSR
*YTU588-012-R*		ATTCATTGGAATGGACGGAG	SSR
*YTU588-013-F*	chr2B_659070427	TGTACATGGATAGCAACGGC	SSR
*YTU588-013-R*		TTTCGTTCACAATGCGGTAA	SSR
*YTU588-014-F*	chr2B_663129913	GGAAGTCAGTGATAGGGGCA	SSR
*YTU588-014-R*		CAAATACTCCCTCCGTTCCA	SSR
*YTU588-015-F*	chr2B_664211170	GAGGGCTAAATGAGCAGCAG	SSR
*YTU588-015-R*		GCCTTCCGTGGACAAGTTTA	SSR
*YTU588-016-F*	chr2B_664211170	TAAACTTGTCCACGGAAGGC	SSR
*YTU588-016-R*		ATGGAGAGGTAGACCCCGAC	SSR
*YTU588-017-F*	chr2B_664221850	CACCAAGATCCGAACCAGAT	SSR
*YTU588-017-R*		ACCTCCTCCGACTTAGCGTT	SSR
*YTU588-018-F*	chr2B_667265948	TATCTAGCCATGGACAGGGG	SSR
*YTU588-018-R*		AAAATCAAATAGCTGAATCATGG	SSR

### Discovery and Classification of Differentially Expressed Genes

**FIGURE 5:**
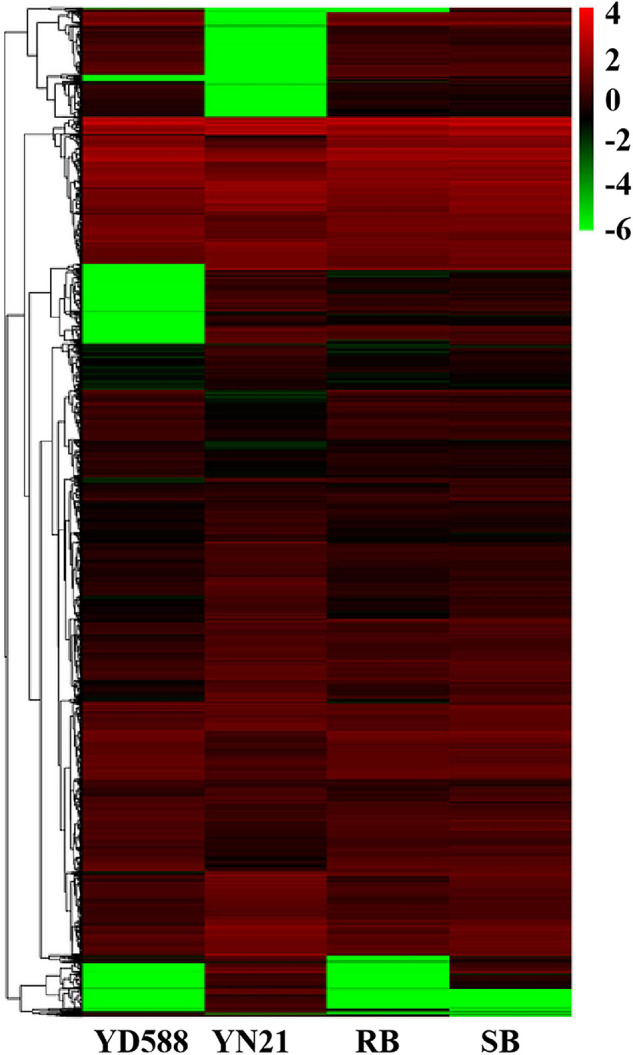
Heat map of the differentially expressed genes (DEGs) between the resistance and susceptible parents YD588 and YN21 and their derived resistance and susceptible bulks from their derived F_2__:__3_ families. RB, resistance bulk; SB, susceptible bulk.

After SNP calling, DEGs were annotated between the two parents and two bulks, respectively. A total of 12,118 DEGs were identified from the parents and bulks (Figure 5). Among them, 10,816 DEGs were identified between parents YD588 and YN21, where 6,305 ones were downregulated and 4,511 ones were upregulated using the expression index of YN21 as a standard (Supplementary Table 4). Furthermore, 3,360 DEGs were detected between the resistance and susceptible bulks, where 2,642 and 718 were downregulated and upregulated, respectively (Supplementary Table 5). Finally, 2,973 DEGs showed a consistent difference between parents and bulks, which can be used for subsequent GO, COG, and KEGG analysis (Supplementary Table 6).

### Gene Ontology, Clusters of Orthologous Group, and Kyoto Encyclopedia of Genes and Genomes Pathway Enrichment Analyses of Candidate Genes

**FIGURE 6:**
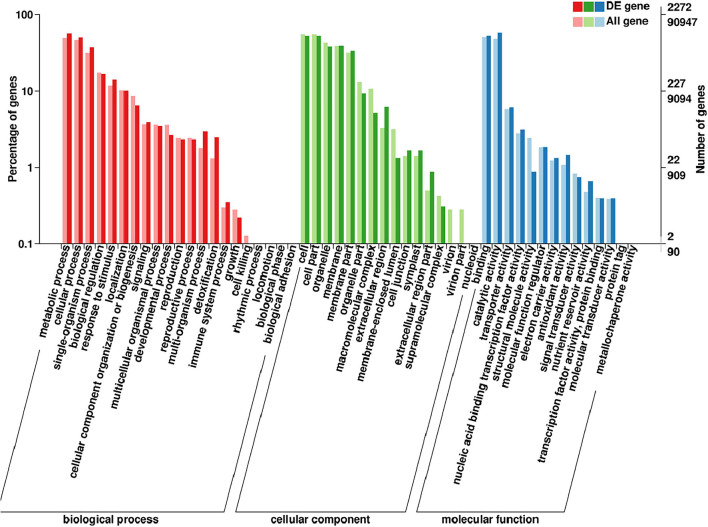
Gene ontology (GO) analysis of the DEGs with consistent differences between the resistance and susceptible parents YD588 and YN21 and their derived resistance and susceptible bulks from their derived F_2__:__3_ families.

Gene ontology database is a standard biological annotation system. DEGs showed a consistent difference between parents, and bulks were classified using the GO analysis. The results indicated that these DEGs were mainly involved in cell components, including cell, membrane, organelle, and cell part; in molecular functions, including catalytic activity, binding, and transporter activity; in biological processes, including metabolic and cellular processes, single-organism processes, and response to stimulus and biological regulation (Figure 6). It is worth noting that the functional classification in response to stimulus in biological progress was significantly enriched, and these genes may directly participate in the disease defense process.

**FIGURE 7:**
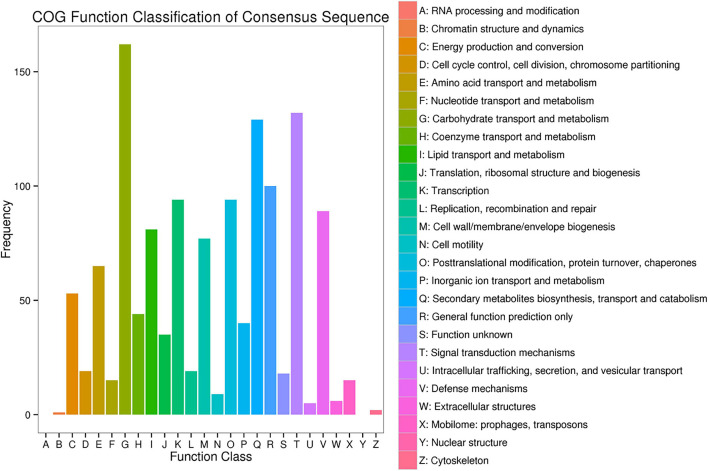
Clusters of orthologous groups (COG) analysis of the DEGs with consistent differences between the resistance and susceptible parents YD588 and YN21 and their derived resistance and susceptible bulks from their derived F_2__:__3_ families.

To further explore the regulatory genes that may relate to powdery mildew, COG analysis and KEGG pathway enrichment analyses were performed on DEGs that showed consistent differences between parents and corresponding bulks (Figure 7). From the data of COG, 0.8% of candidate genes were found to be directly involved in the plant defense mechanism, while most of the candidate genes account for amino acid and carbohydrate transport and metabolism, and DNA duplication, recombination, and repair. This probably indicated that more diverse or alternative metabolism and synthesis processes are being activated during plant immune response, which is consistent with previous studies (Rudd et al., 2015; Verbancic et al., 2018).

**FIGURE 8:**
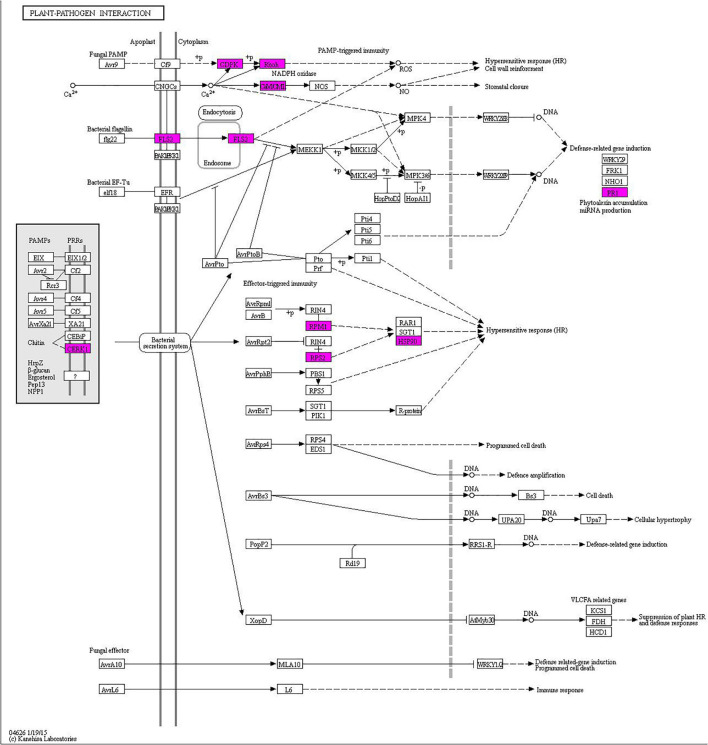
The plant-pathogen interaction pathway enriched from DEGs with consistent differences between the resistance and susceptible parents YD588 and YN21 and their derived resistance and susceptible bulks from their derived F_2__:__3_ families.

Using the KEGG analysis, 115 significantly enriched pathways accounting for 20 categories in cellular processes, environmental processing, genetic information processing, metabolism, and organismal system were found (Supplementary Figure 1 and Supplementary Table 7). In particular, one plant-pathogen interaction pathway was enriched, which can be used to select key regulatory genes for further time-course analysis postinoculation with *Bgt* (Figure 8).

### Expression Analysis for the Disease-Resistance Related Genes

**FIGURE 9:**
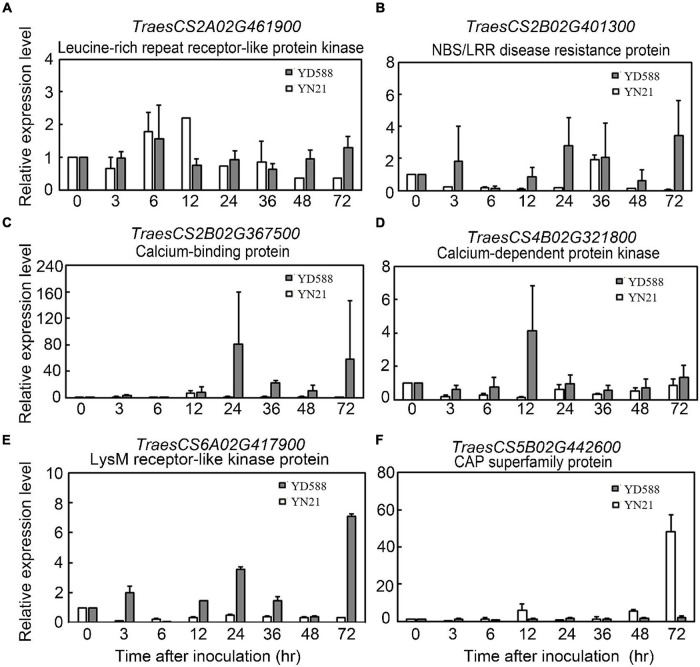
Expression profiles of *TraesCS2A02G461900* **(A)**, *TraesCS2B02G401300* **(B)**, *TraesCS2B02G367500* **(C)**, *TraesCS4B02G321800* **(D)**, *TraesCS6A02G417900* **(E)**, and *TraesCS5B02G442600* **(F)** in each corresponding stage of YD588 and YN21 after *Bgt* infection.

In the enriched plant-pathogen interaction pathway, 23 genes were involved, and the sequence information of one new gene was not clear (Supplementary Table 8). Therefore, we monitored the expression level of the remaining 22 genes in this pathway following *Bgt* inoculation. As a result, six of them showed significant differences between YD588 and YN21 in the time-course analysis following *Bgt* inoculation (Figure 9).

The transcriptional levels of two LRR genes (*TraesCS2A02G461900* and *TraesCS2B02G401300*) were upregulated in YD588 but not in YN21 despite different patterns in the time scale. *TraesCS2B02G401300* was induced at an early stage within 3 h and lasted until 48 and 72 h, while *TraesCS2A02G461900* was significantly upregulated only after 48 h (Figures 9A,B). Two calcium-associated genes *TraesCS4B02G321800* and *TraesCS2B02G367500* were obviously induced at 24 h after inoculation, while the expression of *TraesCS4B02G321800* was elevated from 3 h, and *TraesCS2B02G367500* was induced from 24 h (Figures 9C,D). The transcription of *TraesCS6A02G417900* showed an obvious periodicity, which was stimulated at 3 h, downregulated at 6 h, and peaked two times at 24 and 72 h (Figure 9E). In contrast to the expression trend of the above genes, the transcript of *TraesCS5B02G442600*, one of the pathogenesis-related 1 proteins (CAP) superfamily proteins, was not induced any time after *Bgt* inoculation in YD588 (Figure 9F).

## Discussion

In this study, the wheat breeding line YD588 was proved to be resistant to powdery mildew. Using BSR-Seq and molecular markers, a dominant gene, tentatively designated *PmYD588*, confers the broad-spectrum seedling resistance to different *Bgt* isolates. To preliminarily dissect the resistance mechanism that participated in the resistance process, we also profiled the gene expression pattern after *Bgt* invasion in a wheat breeding line YD588.

Using BSR-Seq, the *R* gene *PmYD588* was first proved to be associated with two adjacent candidate intervals 453,752,054-506,356,791 and 584,117,809-664,221,850 bp in the chromosome arm 2B. Then, using molecular markers derived from the BSR-Seq, it was further located at an interval of 4.2 cM corresponding with the 647.1-656.0 Mb physical interval based on the reference genome of Chinese Spring. Prior to *PmYD588*, ten *Pm* genes, namely, *Pm6* (Wan et al., 2020), *Pm33* (Zhu et al., 2005), *Pm51* (Zhan et al., 2014), *Pm52* (Wu et al., 2019), *Pm63* (Tan et al., 2019), *Pm64* (Zhang et al., 2019), *PmQ* (Li Y. et al., 2020), *PmKN0816* (Wang et al., 2021), *MlZec1* (Mohler et al., 2005), and *MlAB10* (Maxwell et al., 2010) have previously been reported on the chromosome arm 2BL. However, *PmYD588* can be distinguished from *Pm6* (698.3–699.2 Mb), *Pm33* (779.1–784.3 Mb), *Pm51* (709.8–739.4 Mb), *Pm52* (581.0–585.0 Mb), *Pm63* (710.3–723.4 Mb), *Pm64* (699.2–705.5 Mb), *PmQ* (710.7–715.0 Mb), *PmKN0816* (700.4–710.3 Mb), *MlZec1* (796.7–780.0 Mb), and *MlAB10* (796.7–780.0 Mb) in its physical interval, suggesting *PmYD588* is most likely a new *Pm* gene. In spite of this, more evidence is still needed in the future to clarify the relationship of these *Pm* genes on the chromosome arm 2BL, such as mutual allelism tests and even cloning of these genes.

In the process of plant disease resistance, the functional exhibition not only depends on the *R* gene but also regulates a mass of genes in the innate immune system of the plants. To further describe the potential resistance mechanism in the YD588, we enriched a plant-pathogen interaction pathway that may be related to the functional exhibition of the *R* gene and selected 22 genes for time-course analysis postinoculation with *Bgt*.

Among the 22 candidate genes in the plant-pathogen interaction pathway, five genes were described as LRR-RLK family protein and two genes labeled as NBS-LRR disease resistance protein homolog. These genes with the LRR domain accounted for ∼32% of all candidate genes. It has been reported that pattern-recognition receptor-like kinases (RLKs) or proteins (RLPs) possessing LRR domain activate effector-triggered immunity (ETI) in several plant species (Saur et al., 2000; Cui et al., 2015; Pruitt et al., 2015; Couto and Zipfel, 2016; Mott et al., 2016; Yan et al., 2018; Yuan et al., 2021). In our study, the transcriptional levels of two LRR genes (*TraesCS2A02G461900* and *TraesCS2B02G401300*) were upregulated in YD588 but not in YN21 because of different patterns. At the beginning of *Bgt* inoculation, there was no significant expression difference of *TraesCS2A02G461900* in YD588 and YN21, but after 48 h of treatment, the expression was significantly upregulated only in YD588. In contrast, *TraesCS2B02G401300* was induced at an early stage within 3 h and lasted for 48 and 72 h. The difference in response time of these two genes may be due to the direct or indirect regulation of the two genes in the process of powdery mildew resistance, and considering *TraesCS2A02G461900* was a kinase, it may be functioned by phosphorylating other ligands while *TraesCS2B02G401300* was not (Figures 9A,B).

Calcium-binding protein, calcium-dependent protein kinase (CDPKs), or calmodulin protein account for about 27% of the 22 selected genes in the in the plant-pathogen interaction pathway (6/22). Calcium-binding protein and CDPKs are known to play pivotal roles during abiotic and biotic stress responses (Coca and Segundo, 2010; Feng et al., 2011; Asano et al., 2012). It has been reported that CDPKs regulated host cell entry in barley powdery mildew resistance (Freymark et al., 2007), and calcium-binding protein *TaCab1* was induced during stripe rust infection (Feng et al., 2011). In our result, the transcriptional levels of *TraesCS4B02G321800* described as CDPK and *TraesCS2B02G367500* encoding a calcium-binding protein were detected. These two genes were both obviously induced 24 h after inoculation, while the expression of *TraesCS4B02G321800* was elevated from 3 h and *TraesCS2B02G367500* was induced from 24 h (Figures 9C,D).

In our remaining candidate genes, *TraesCS6A02G417900* encoded a Lysin Motif (LysM) receptor-like kinase protein. LysMs have been reported to bind or percept N-acetylglucosamine (GlcNAc)-containing molecules produced by microorganisms and play a vital role in the plant-pathogen interaction (Radutoiu et al., 2003; Buendia et al., 2018). The transcription of *TraesCS6A02G417900* was stimulated at an early time after *Bgt* inoculation (3 h) and then downregulated at 6 h. Subsequently, two peaks emerged in the expression trend, one at 24 h and another at 72 h (Figure 9E). The fluctuant expression of *TraesCS6A02G417900* implied the periodical function during resistance response.

Cysteine-rich secretory proteins, antigen 5, and pathogenesis-related 1 protein (CAP) superfamily protein was another relatively large family protein in our candidate pool, accounting for ∼23% (5/22). CAP superfamily is involved in cancer and immune defense in animals (Gibbs et al., 2008). Small peptides derived from the CAP superfamily play important roles in salt stress and immune signaling in *Arabidopsis* and tomato, respectively (Chien et al., 2015). The transcript of *TraesCS5B02G442600*, one of the CAP superfamily proteins, was monitored in the time course following *Bgt* inoculation. However, induced expression was not observed any time after *Bgt* inoculation in YD588. In contrast, high-level expression was detected in YN21 at 72 h. Considering the previous studies in other plant species, we concluded that CAP superfamily protein might not function directly and might be activated after splicing or other modification processing.

## Conclusion

In conclusion, using a resistant wheat breeding line YD588, we identified a powdery mildew resistance gene PmYD588, investigated the resistance expression profile to powdery mildew, and further selected several key candidate genes for time-course analysis postinoculation with *Bgt*. Our study can be valuable for enhancing the genetic diversity, understanding resistance pathways, and preliminarily dissecting the expression profiles after *Bgt* invasion.

## Data Availability Statement

The datasets presented in this study can be found in online repositories. The names of the repository/repositories and accession number(s) can be found below: NCBI Sequence Read Archive (SRA), PRJNA761248>.

## Author Contributions

PM, CL, and BW conceived the research. PM, BW, LW, YX, XZ, and WW performed the experiments. PM and BW analyzed the data. PM and HX performed phenotypic assessments. BW and PM wrote the manuscript. All authors read and approved the final manuscript.

## Conflict of Interest

The authors declare that the research was conducted in the absence of any commercial or financial relationships that could be construed as a potential conflict of interest.

## Publisher’s Note

All claims expressed in this article are solely those of the authors and do not necessarily represent those of their affiliated organizations, or those of the publisher, the editors and the reviewers. Any product that may be evaluated in this article, or claim that may be made by its manufacturer, is not guaranteed or endorsed by the publisher.

## Footnotes

1
http://www.wheatgenome.org/

2
http://www.ncbi.nlm.nih.gov/COG

3
https://www.kegg.jp/kegg/pathway.html
