# Supplementary material for: Bulked Segregant RNA-Seq Provides Distinctive Expression Profile Against Powdery Mildew in the Wheat Genotype YD588
Source: Front Plant Sci. 2021 Dec 3;12:764978. doi: 10.3389/fpls.2021.764978 (PMC8677838; doi:10.3389/fpls.2021.764978)
Supplement: Supplementary file 2 [file Table_2.DOCX]

**Table S2.** Data summary of RNA-seq for YD588, Yannong 21 (YN21) and their derived resistant and susceptible bulks obtained from 40 homozygous resistant and 40 homozygous susceptible F_2:3_ families, respectively.

| Samples | Total base pairs | Clean reads | GC content of clean reads (%) | Clean reads Q30 (%) | Genome map rate (%) |
| --- | --- | --- | --- | --- | --- |
| YD588 | 13,352,701,160 | 44,657,220 | 56.12 | 94.89 | 86.28 |
| YN21 | 10,434,996,726 | 34,873,857 | 52.49 | 94.87 | 61.88 |
| Resistance bulk | 28,599,294,802 | 95,638,544 | 55.80 | 94.84 | 86.43 |
| Susceptible bulk | 24,871,361,832 | 83,147,454 | 55.88 | 95.01 | 81.34 |
