# Supplementary material for: Bulked Segregant RNA-Seq Provides Distinctive Expression Profile Against Powdery Mildew in the Wheat Genotype YD588
Source: Front Plant Sci. 2021 Dec 3;12:764978. doi: 10.3389/fpls.2021.764978 (PMC8677838; doi:10.3389/fpls.2021.764978)
Supplement: Supplementary file 8 [file Table_8.DOCX]

**Table S8**. The candidate genes in the plant-pathogen interaction pathway and primers designed based on their coding sequences for RT-qPCR.

| Gene ID | Functional annotation | Forward primer | Reverse primer |
| --- | --- | --- | --- |
| TraesCS2A02G461900 | Leucine-rich repeat receptor-like protein kinase | GCTGCTGGCGTTCAAGAAG | GAGCTGGAGCGTGGAGAT |
| TraesCS2B02G401300 | Disease resistance protein; NBS/LRR disease resistance protein | GTATCCAATTCAGAGGCGCT | TCTGACTGTTGATATCTGGAGTTG |
| TraesCS2B02G367500 | Calcium-binding protein | CTCCTACATCCCGACCAGC | CCAGCGAGTCCTCCAGCT |
| TraesCS4B02G321800 | Calcium-dependent protein kinase | GCAGCCCGTTCTATGTTGC | CTCTGAAGGGCAATTGCATAG |
| TraesCS6A02G417900 | LysM receptor-like kinase protein | TCACGAAGAACACGAACATCAC | GTGAGGTTGATGTAGTTGCTGG |
| TraesCS5B02G442600 | CAP (Cysteine-rich secretory proteins Antigen 5 and Pathogenesis-related 1 protein) superfamily protein | GCGACTGCCAGCTGATAC | GATGGCCGTCGAGTCAC |
| TraesCS1A02G308700 | Calmodulin 7 | GACGAGTGCAAGGAGGTGTT | CCAGTCCCTTGGCCGATA |
| TraesCS2A02G461800 | Leucine-rich receptor-like protein kinase family protein | AGCTCCTGGACCTCAGCAAC | GTCGAGATTGTTCATAAAGGCC |
| TraesCS2B02G435600 | NBS-LRR disease resistance protein homologue | AGAACTTCACTACGGATGACCTTC | TCTTGACTGCGTGTTGTGATG |
| TraesCS2B02G483400 | Receptor kinase | ACCTCGGCAACAACTCCC | CGTGAGCTTGGCGAATGA |
| TraesCS2B02G483500 | Leucine-rich repeat receptor-like protein kinase | TTTATAAACAATCTCGACGGCG | GCCTCCAGGTTGACGAGC |
| TraesCS2D02G347500 | Calcium-binding protein | CGAGCTGTTCGACCGGAA | CATGTCCTCGTCCTCGTCCT |
| TraesCS2D02G462000 | Leucine-rich receptor-like protein kinase family protein | AATCTCCAGAAGCTCGTCGTC | AAGTTGTTCCACGAAAGGTCC |
| TraesCS2D02G462100 | Leucine-rich repeat receptor-like protein kinase | ACCTCGGCAACAATTCCCT | GATCGAGCCGGACAGCTT |
| TraesCS3B02G314000 | Respiratory burst oxidase-F-like protein | GCACAACTACCTCACAAGCG | AGCCTTGCTCACGTGTCA |
| TraesCS3D02G484900 | Calcium binding protein | TGTTCCGCAAGTTCGACG | ACACCTTGAAGGCCAGCC |
| TraesCS4B02G091600 | NBS-LRR disease resistance protein homologue | ACTTCTATAGTTGACAGGTGCCGT | TTTCAGGTGACATGTGGTAGTCTT |
| TraesCS5A02G439700 | CAP (Cysteine-rich secretory proteins Antigen 5 and Pathogenesis-related 1 protein) superfamily protein | ATGGAGTACTCGCCGAAGC | GGAGTATGTATCAGCTGGCAGT |
| TraesCS5A02G439800 | CAP (Cysteine-rich secretory proteins Antigen 5 and Pathogenesis-related 1 protein) superfamily protein | GCAGGACTTCGTGGACCC | CTTCTCCGATACCCACGCTT |
| TraesCS5A02G490200 | Calcium dependent protein kinase | CTGCAATGAACAATCTACAGAAATT | CCAACTAGCTCACTGAAAACCT |
| TraesCS5D02G446800 | CAP (Cysteine-rich secretory proteins Antigen 5 and Pathogenesis-related 1 protein) superfamily protein | AACTCGCCGCAGGACTTC | CCGTGGTCGTAGTACTGCTTCT |
| TraesCS5D02G446900 | CAP (Cysteine-rich secretory proteins Antigen 5 and Pathogenesis-related 1 protein) superfamily protein | ATGGAGTACTCGCCGAAGATAG | CGAATGTACCAGCTGGCAG |
| Tubulin | housekeeping gene | CAAGGAGGTGGACGAGCAGATG | GACTTGACGTTGTTGGGGATCCA |
