# Supplementary figures and images for: Bulked Segregant RNA-Seq Provides Distinctive Expression Profile Against Powdery Mildew in the Wheat Genotype YD588
Source: Front Plant Sci. 2021 Dec 3;12:764978. doi: 10.3389/fpls.2021.764978 (PMC8677838; doi:10.3389/fpls.2021.764978)

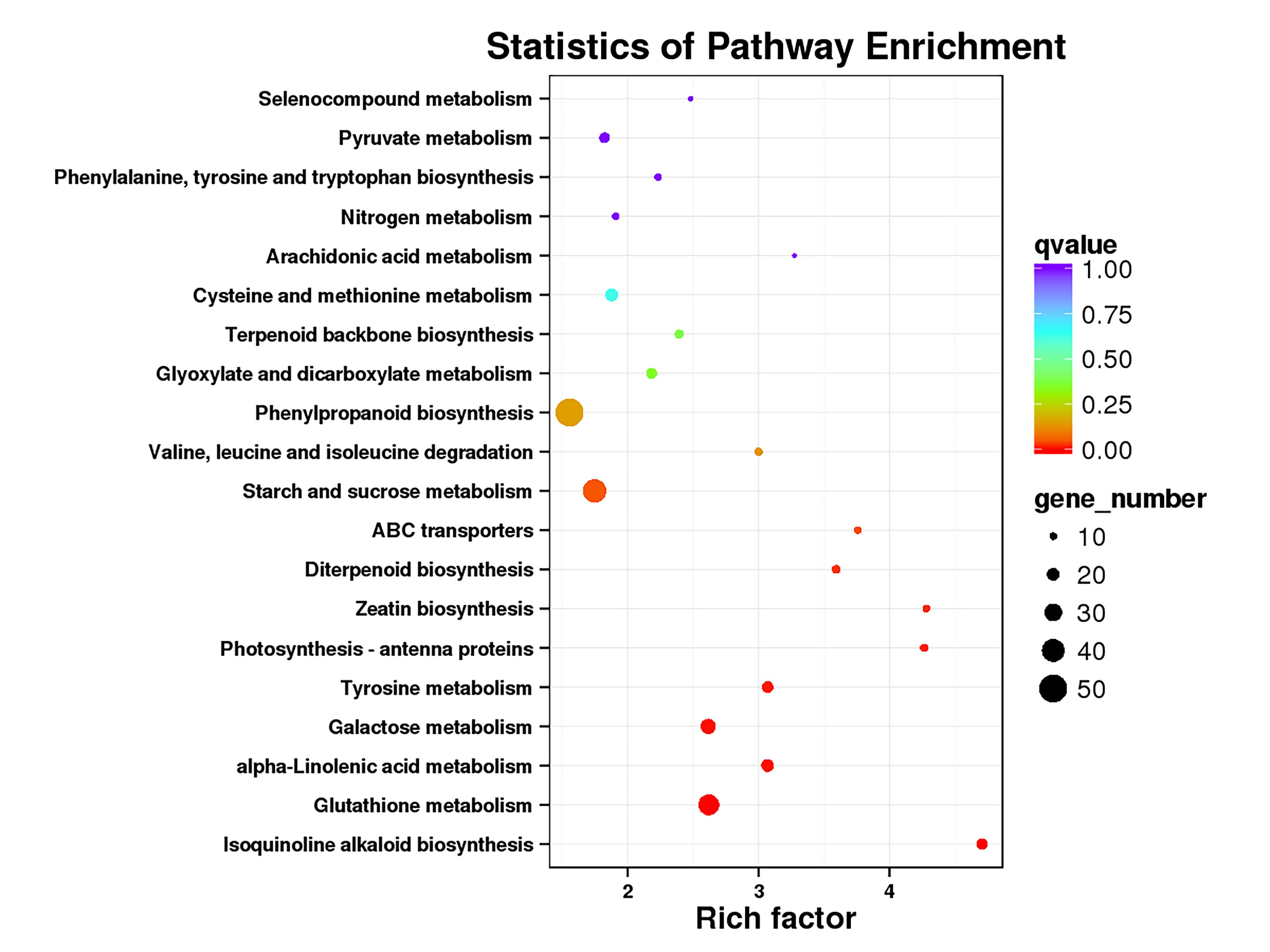

Supplement: Supplementary file 9 [file Image_1.JPEG]
